# Pathologic examination of the placenta and its benefits in treatment plan or follow-up of patients: a cross-sectional study

**DOI:** 10.1186/s40001-022-00743-7

**Published:** 2022-07-11

**Authors:** Setareh Akhavan, Sedigheh Borna, Alireza Abdollahi, Mamak Shariat, Narges Zamani

**Affiliations:** 1 Department of Gynecologic Oncology, Vali-Asr Hospital, Tehran University of Medical Sciences, Imam Khomeini Hospital Complex (IKHC) , Keshavarz Blvd, Tehran, Iran; 2 Department of Perinatalogy, Vali-e-Asr Hospital, Tehran University of Medical Sciences, Tehran, Iran; 3 Department of Pathology, Imam Hospital Complex, Tehran University of Medical Sciences, Tehran, Iran; 4 Maternal, Fetal & Neonatal Research Center-Breastfeeding Research Center, Tehran University of Medical Sciences Tehran, Tehran, Iran

**Keywords:** Follow-up, Pathology, Placenta, Treatment plan

## Abstract

**Background:**

The placental examination provides important information about the effect of maternal abnormalities on the placenta or the cause of preterm delivery, fetal growth restriction, or fetal neurodevelopmental damage. In this study, the frequency of placental pathologies of patients in a tertiary hospital was investigated.

**Methods:**

In this longitudinal and cross-sectional study, all removed placentas after any type of pregnancy termination referred to a pathological examination, within 1 year (2019–2020). All placentas were examined macroscopically and microscopically by two pathologists.

**Results:**

Unfortunately, because of the COVID-19 pandemic, the number of pregnant women in our hospital declined. A total of 258 placentas were examined. The type of delivery in 193 cases (79.4%) was cesarean section and 50 cases (20.6%) had a vaginal delivery. In the pathological assessment of placentas, 238 (92.2%) cases were normal and 20 cases (7.8%) were abnormal. Infarct and chorioamnionitis were the pathologies with higher frequencies (4.3% and 2.7%, respectively). Intra-uterine fetal death (*p* = 0.701), preeclampsia (*p* = 0.51) had no significant difference was seen in normal and abnormal placentas. Maternal age (*p* = 0.83), gestational age based on the last menstrual period (*p* = 0.38), and gestational age based on the first ultrasound (*p* = 0.78) did not show a significant relationship with any of the pathological complications categories.

**Conclusions:**

Pathological examination of the placenta from all live-birth deliveries is not worthwhile, and it’s recommended to modify the guidelines as to when the placenta is submitted for pathological evaluation.

## Background

The placenta is an embryonic organ consisting of the umbilical cord, chorionic and amniotic membranes, and the parenchyma. Maternal or fetal abnormalities may cause placental abruption, because the mother and fetus intersect at this point. On the other hand, early placental abnormalities can affect the health of both mother and fetus. The placental examination provides valuable information about the effect of maternal abnormalities on the placenta or the cause of preterm delivery, fetal growth restriction, or fetal neurodevelopmental damage. The placental examination is an essential component of autopsy in cases of fetal or infant death [1–3].

In the following cases, pathological examination of the placenta is recommended: (1) stillbirth (present or past), (2) infant resuscitation or hospitalization in the NICU, (3) pre-term or post-term delivery, (4) pregnancy with twins or multiples, (5) Apgar score below 7, (6) obstetric complications (e.g., chorioamnionitis, preterm labor, preeclampsia, cholestasis, intolerance of the baby to labor, intrauterine or postpartum hemorrhage, thick meconium, severe polyhydramnios or oligohydramnios), (7) observation of gross abnormality in the placenta (such as abnormal color, mass, short or long umbilical cord, abnormal membranes), (8) fetal or neonatal abnormalities or hydrops; and (9) obvious maternal disorders and diseases (such as diabetes, obesity, hypertension, smoking, alcohol, and addictive drugs) thyroid disease, malignant placental neoplasm, fever/infection, abnormality or scarring of the uterus [4–8].

Any tissue that removes from the human body is indicated for pathological examination, but only cases with indications are evaluated in many hospitals [9]. The American College of Pathologists also stated that at the Perinatal Services Level 3 Center, 20% of couples undergo a pathological examination, while 50% should be evaluated and offer several indications, and thus many cases; which were previously hidden from view, are identified [10]. Pathological evaluation of the placenta can help us to understand the pathophysiology of many pregnancy diseases and even to achieve preventive ways.

One of the most important and vital cases of abnormal placentas that requires early diagnosis and timely action is gestational trophoblastic diseases (GTD), which includes a wide range of proliferative disorders of placental trophoblastic tissue, including moles [11]. Complete and incomplete hydatidiform that are premalignant and its invasive types include gestational trophoblastic neoplasia, including invasive mole, choriocarcinoma, placental site trophoblastic tumor (PSTT), and epithelioid trophoblastic tumor (ETT) [1].

Due to the importance of placental pathologies and the complications it causes, in this study, the frequency of placental pathological manifestations in deliveries of Vali-Asr Hospital—Imam Khomeini Hospital Complex in Tehran was investigated.

## Materials and methods

This longitudinal and cross-sectional descriptive study was performed in the delivery block of Vali-Asr Hospital, Tehran University of Medical Sciences, Tehran, Iran. This study was performed According to the principles of the Declaration of Helsinki. Approval was granted by the Ethics Committee of Tehran University of Medical Sciences (TUMS) (Ethical code#: IR.TUMS.VCR.REC.1397.994).

During 1 year, all placenta samples were sent to the pathology laboratory for examination within 2 h after the end of pregnancy. The placenta was kept in a 10% formalin solution and delivered to the pathology department as soon as possible.

### Data collection

In the laboratory, the following measures were taken to prepare placenta samples: the placenta samples were first examined macroscopically and then microscopically in the pathology department. Macroscopic examination in terms of maternal surface, umbilical length, hematoma, membrane transparency, and lobulation was performed for all samples. In the microscopic examination, after preparing H&E slides, all of them were examined for fibrosis, knot syncytial, trophoblastic cells, villi, fibroid deposit, inflammation, necrosis, vessels, and calcification. Data were recorded in a researcher-developed questionnaire.

### Statistical analysis

Data were entered in SPSS (version 20.0 for Windows; IBM SPSS Statistics, Armonk, NY, USA), and analyzed based on objectives. Descriptive statistics were expressed as number and percentage (valid percent) for qualitative variables and as mean and standard deviation for quantitative variables. In analytical statistics, comparisons between two groups and several groups for qualitative variables were performed using the chi-square test. Mann–Whitney test (compared between two groups) and Kruskal–Wallis test (compared between several groups) were performed for variables with abnormal data distribution. Significance levels were considered less than 0.05.

## Results

Unfortunately, because of the COVID-19 pandemic, the number of pregnant women in our hospital were declined. A total of 258 placentas were examined. The type of delivery in 193 cases (79.4%) was cesarean section and 50 cases (20.6%) had a vaginal delivery. Demographic information and quantitative variables are listed in Table 1.

**Table 1:** Demographic characteristics of mothers

Variable	Mean ± SD^a^
Mother’s age (year)	28.48 ± 6.25
Pregnancy	2.43 ± 2.64
Delivery order	1.05 ± 0.93
Abortion	0.417 ± 0.72
Alive child	0.94 ± 0.95
Gestational age (LMP)^b^ (weeks)	35.40 ± 7.83
Gestational age (ultrasound) (weeks)	36.03 ± 6.76
	114.56 ± 11.50
Temperature (°C)	37.89 ± 8.58
PR^c^	86.48 ± 6.89
RR^d^	18.27 ± 1.81
BHCG 60 days after delivery (mIu/ml)	1.24 ± 14.49

^a^Standard deviation

^b^Last menstrual period

^c^Pulse rate

^d^Respiratory rate

There was no history of the disease in 181 cases (70.2%) and hypothyroidism was the most common previous history of the disease (10 cases, 3.9%). In the pathological assessment of placentas, 238 (92.2%) cases were normal and 20 cases (7.8%) were abnormal. The frequency of complications is listed in Table 2.

**Table 2:** Abnormal placental complications in pathological assessment

Complication	Frequency	Percent
Vascular abnormality	1	0.4
Inflammation	1	0.4
Inflammation around the arteries	1	0.4
Infarct	6	2.3
Infarct and inflammation	2	0.8
Infarct and accreta placenta	1	0.4
Infarct and chorioamnionitis	2	0.8
Chorioamnionitis	3	1.2
Chorioamnionitis and vascular abnormality	1	0.4
Chorioamnionitis and choriocarcinoma	1	0.4
Chorioamniotic	1	0.4

Infarct and chorioamnionitis were the pathologies with higher frequencies (4.3% and 2.7%, respectively). The relationship between each category of pathology with IUFD and preeclampsia is shown in Table 3. In addition, the correlation between maternal age, gestational age based on the last menstrual period (LMP), and gestational age based on the first ultrasound with each category of placental pathologies are presented in Table 4.

**Table 3:** Correlation between each pathology category and some maternal and complications

Variable	Normal	Vascular abnormality	Chorio-amnionitis	Infarct	Chorio-carcinoma	*p* value
Qualitative variables						
IUFD^a^						
Yes *n* (%)	8 (3.1)	0	1 (0.4)	0	0	0.547
No *n* (%)	230 (89.1)	1 (0.4)	6 (2.3)	11 (4.2)	1 (0.4)	
Preeclampsia						
Yes *n* (%)	5 (2.0)	0	0	0	0	0.980
No *n* (%)	238 (92.2)	1 (0.4)	7 (2.7)	11 (4.2)	1 (04)	

^a^Intra-uterine fetal death

**Table 4:** Correlation between each some maternal and neonatal variables and complications

Variable		Normal	Vascular abnormality	Chorio-amnionitis	Infarct	Chorio-carcinoma	*p* value
Quantitative variables							
Maternal age	*n* (Mean ± SD)	208 28.6 ± 6.32	1 27.0	5 28.2 ± 7.3	10 26.4 ± 5.1	1 28	0.831
GA^a^/LMP^b^	*n* (Mean ± SD)	181 35.5 ± 7.7	1 40	5 30.9 ± 11.6	8 37.9 ± 2.6	1 15	0.383
GA/ Ultrasound	*n* (Mean ± SD)	127 36.1 ± 6.7	1 39.5	3 32.1 ± 12.2	4 37.3 ± 2.6	–	0.786

^a^Gestational age

^b^Last menstrual period

Regarding IUFD, no significant difference was seen in normal and abnormal placentas (3.5% of normal placentas and 5% of abnormal ones had IUFD *(p* = 0.701)). In normal placentas, 5 cases (2.1%) were preeclamptic and in placentas with abnormal pathology, no case of preeclampsia was observed, and there was no significant difference in terms of preeclampsia in normal or abnormal cases of placental pathology (*p* = 0.51). Maternal age (*p* = 0.83), gestational age based on LMP (*p* = 0.38), and gestational age based on the first ultrasound (*p *= 0.78) did not show a significant relationship with any of the pathological complications categories.

## Discussion

Histopathological examination of the placenta can provide valuable insights into a variety of pregnancy complications. A complete examination of the placenta and umbilical cord by a perinatal pathologist is a necessity to care for the adverse consequences of pregnancy [12]. For example, it has been reported that 25% of stillbirths are due to pathological problems of the placenta and umbilical cord [13]. The present study is one of the few studies in this field that has examined 258 placentas pathologically in a year. Among them, 193 cases (79.4%) had a cesarean delivery and 50 cases (20.6%) had a vaginal delivery; 92.2% of them were normal, and 7.8% were abnormal.

The infarct of the placenta is a macroscopic focal parenchymal lesion that shows necrosis and approaching villous microscopically. Although placental infarction is common in the later stages of pregnancy, a variety of cases have been reported in different stages of pregnancy [14]. According to the results of the present study, placental infarction had the highest frequency among cases of placental pathology (4.3% of the total of 7.8% of complicated placentas). Placental lesions, including placental infarction, are associated with fetal and neonatal mortality, and complications, and are one of the most common placental complications. When premature, focal, or diffuse infarction occurs, it is associated with severe preeclampsia, IUGR, and even fetal death [15].

Our findings showed that there was no significant difference between IUFD and preeclampsia in placentas with normal and abnormal pathology. However, it has been reported that maternal vascular abnormal perfusion is more common in premature and post-term pregnancy (23% and 5%) than in uncomplicated deliveries [16]. On the other hand, IUFD has been revealed as the main consequence of pregnancies complicated by preeclampsia. Placental infarction was seen in more than 5% of IUFD placentas with infarction and cases of small for gestational age (SGA) [17].

Loverro and et al. in 2022 showed that although many reports said that examination of the placenta provided information which important for the management of pregnancy, it still presents unresolved problems because of the pathologic aspect in normal pregnancy [18].

The variables of maternal age, gestational age based on LMP, and gestational age based on the first ultrasound did not show significant differences in placentas with normal and abnormal pathology. Past studies have shown that fetal vascular malformation and delayed puberty are associated with increasing maternal age during pregnancy [17].

Choriocarcinoma is most often seen with complete mole, ectopic pregnancy, non-molar intrauterine abortion, and unusually with partial mole. Choriocarcinoma with or after a “normal” pregnancy is very rare [19]. Intra-placental choriocarcinoma is a focal neoplastic proliferation of placental villi trophoblasts that is a rare type of gestational choriocarcinoma, but because of its rarity, available information is still limited, besides this information is available from individual case reports or series of small cases [20]. Among the studied placentas, one case (0.4%) had choriocarcinoma. According to the results of the study, macroscopic examination of the placenta with choriocarcinoma is not significant and only small abnormal lesions are seen in it, which are thought to be new infarcts or intervillous thrombosis, and histological examination helps to diagnose this lesion [21].

This study had some limitations, such as problems in sending the placenta to the laboratory quickly. To solve this problem, proper coordination and continuous monitoring were performed during the delivery emergency, and samples were sent with appropriate conditions and time to Vali-Asr Hospital laboratory. Finally, the small sample size of the study made it possible to reduce the study power and, therefore, further studies such as multicenter research with larger sample size is needed.

## Conclusions

Pathological examination of the placenta is very important for early examination and finding cases that may have a poor prognosis of mother and infant; However, according to the results of the present study, since there was no significant difference in placental pathology in cases of IUFD and preeclampsia in mothers of different ages and gestational ages, so Pathologic examination of the placenta from the all live birth deliveries is not worthwhile and due to the cost to the Health System is recommended only in suspicious and high-risk cases. multicenter cohort studies with large sample sizes are needed to confirm these results.

## Data Availability

All data generated or analysed during this study are available for review by the Editor-in-Chief of this journal on request.

## Abbreviations

NICU: Neonatal intensive care unit
GTD: Gestational trophoblastic diseases
PSTT: Placental site trophoblastic tumor
EET: Epithelioid trophoblastic tumor
IUFD: Intrauterine fetal demise
LMP: The last menstrual period
IUGR: Intrauterine growth restriction
SGA: Small for gestational age
